# Hyperthermic intraperitoneal chemotherapy (HIPEC) plus systemic chemotherapy versus systemic chemotherapy alone in locally advanced gastric cancer after D2 radical resection: a randomized-controlled study

**DOI:** 10.1007/s00432-023-05019-z

**Published:** 2023-07-01

**Authors:** Pengfei Yu, Xingmao Huang, Ling Huang, Gaiguo Dai, Qi Xu, Jingquan Fang, Zeyao Ye, Tengjiao Chai, Yian Du

**Affiliations:** 1 Department of Gastric Surgery, Zhejiang Cancer Hospital, Hangzhou Institute of Medicine (HIM), Chinese Academy of Sciences, Hangzhou, 310022 Zhejiang China; 2 Department of Medical Oncology, Zhejiang Cancer Hospital, Hangzhou Institute of Medicine (HIM), Chinese Academy of Sciences, Hangzhou, 310022 Zhejiang China

**Keywords:** Gastric cancer, Chemotherapy, Hyperthermic intraperitoneal chemotherapy (HIPEC), Peritoneal metastasis, Prognosis

## Abstract

**Background:**

Currently, there is a lack of an effective strategy for the prevention of peritoneal metastasis (PM) from locally advanced gastric cancer (AGC). This randomized-controlled study aimed to evaluate the outcome of D2 radical resection with hyperthermic intraperitoneal chemotherapy (HIPEC) plus systemic chemotherapy versus systemic chemotherapy alone in locally AGC patients.

**Methods:**

All enrolled patients were randomly assigned to receive HIPEC plus systemic chemotherapy (HIPEC group) or systemic chemotherapy alone (non-HIPEC group) after radical gastrectomy. HIPEC was performed intraperitoneally with cisplatin (40 mg/m^2^) within 72 h after surgery, while systemic chemotherapy based on the SOX regimen (S-1 combined with oxaliplatin) was administered 4–6 weeks after radical surgery. Patterns of recurrence, adverse events, 3-year disease-free survival (DFS), and overall survival (OS) were analyzed.

**Results:**

A total of 134 patients were enrolled in the present study. The 3-year DFS rate was 73.8% in the HIPEC group, which was significantly higher than that in the non-HIPEC group (61.2%, *P = *0.031). The 3-year OS rate was 73.9% in the HIPEC group and 77.6% in the non-HIPEC group, with no significant difference (*P = *0.737). PM was the most common distant metastasis in both groups. The occurrence rate of PM in the HIPEC group was statistically lower than that in the non-HIPEC group (20.9% vs. 40.3%, *P = *0.015). Grade 3 or 4 adverse events occurred in 19 (14.2%) patients, and there was no significant difference between the two groups.

**Conclusion:**

Radical surgery followed by HIPEC combined with systemic chemotherapy is a safe and feasible strategy for locally AGC patients and could effectively improve DFS and reduce the occurrence of PM. However, more prospective randomized studies with a large sample size are warranted.

**Trial registration:**

This study was registered with www.medresman.org.cn as ChiCTR2200055966 on 10/12/2016.

## Background

Gastric cancer (GC) is one of the most common malignancies worldwide, ranking as the fourth leading cause of cancer-related deaths, and it has a poor 5-year survival rate, which is mainly caused by tumor progression and recurrence (Song et al. 2017; Smyth et al. 2020; Sung et al. 2021). The peritoneum is the most common metastasis site in GC patients after curative resection, especially for patients with serosal invasion or lymphatic metastasis, and exfoliation of free cancer cells in the abdominal cavity is the main cause of peritoneal metastasis (PM) (Montori et al. 2014; Bieri et al. 2015; Coccolini et al. 2016). As reported by the previous studies, more than 50% of GC patients experience PM after radical surgery, causing a poor prognosis of these patients with a median survival time of less than 6 months (Sugarbaker et al. 2003; Thomassen et al. 2014). To date, there is a lack of consensus on preventing PM and improving the prognosis of patients with locally advanced gastric cancer (AGC).

Hyperthermic intraperitoneal chemotherapy (HIPEC) provides direct delivery of concentrated, heated chemotherapeutic drugs into the abdominal cavity, maintaining the thermo-thermal effect and increasing the exposure of cancer cells to chemotherapy to improve the anti-tumor efficacy (Cai et al. 2018; Dodson et al. 2018). The combination of HIPEC and systemic chemotherapy is emerging as a potential regimen for the prevention and treatment of PM in various malignancies (Costa et al. 2012; van Driel et al. 2018; Brenkman et al. 2019; Ceelen 2019). The effect of HIPEC in preventing tumor recurrence and metastasis of AGC remains controversial. Some studies reported that GC patients treated with HIPEC and systemic chemotherapy had a significantly higher recurrence-free survival than patients who did not receive this treatment (Hirose et al. 1999; Zhibing et al. 2013). However, other studies indicated that HIPEC failed to improve the OS and DFS of GC patients (Kunisaki et al. 2002; Diniz et al. 2020). Thus, the adoption and efficacy of HIPEC in GC patients need further study.

This prospective, randomized, controlled study was performed to determine the clinical benefit of the combination of HIPEC and systemic chemotherapy on locally AGC patients after radical resection and provide an effective treatment strategy for these patients.

## Materials and methods

### Study design

This prospective, randomized, controlled study (registration: www.medresman.org.cn; #ChiCTR2200055966) was performed at Zhejiang Cancer Hospital from January 2017 to January 2021. The study was approved by the Institutional Ethics Review Board of Zhejiang Cancer Hospital (Approval No. IRB-2016–157). Written informed consent was obtained from each enrolled patient. The primary endpoint was the 3-year OS rate, and the secondary endpoints were the 3-year DFS rate and safety.

The enrollment criteria were as follows: (1) first diagnosed AGC patients with T3 ~ T4b confirmed by histologic evidence of resected specimens according to the seventh edition of the TNM classification for gastric cancer (Sobin and Ch 2010); (2) without distant metastasis; (3) age between 18 and 75 years; (4) did not receive any preoperative treatment, such as preoperative chemotherapy or radiotherapy; (5) Eastern Cooperative Oncology Group (ECOG) performance status of 0–1; (6) with white blood cells ≥ 3.5 × 10^9^/L, neutrophils ≥ 1.5 × 10^9^/L, platelets ≥ 100 × 10^9^/L, serum total bilirubin ≤ 1.5-fold of the upper limits of the normal ranges (ULNS), serum creatinine ≤ 1.2-fold ULNS, serum aspartate transaminase (AST), and alanine transaminase (ALT) level ≤ 1.5-fold the ULNS. Patients with positive cytology were excluded from the study.

### Treatment

**Fig. 1:**
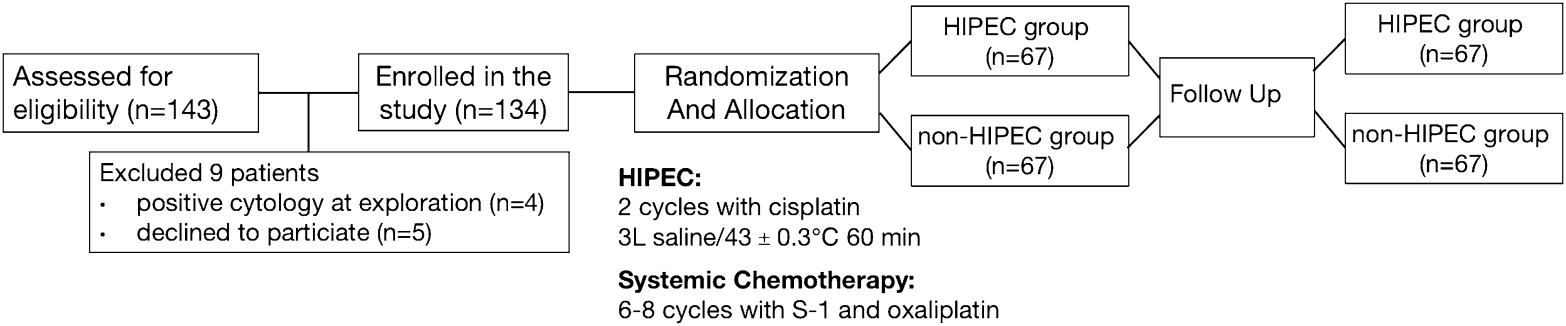
Treatment schedule for advanced gastric cancer patients randomized into the HIPEC group and the non-HPIEC group

All patients were randomly assigned to the HIPEC group or non-HIPEC group after radical gastrectomy using a web response system. Patients in the HIPEC group received HIPEC treatment and systemic chemotherapy, while patients in the non-HIPEC group received only systemic chemotherapy. The treatment schedule of this study is shown in Fig. 1.

I. Surgical treatment

All patients received open or laparoscopic surgery, and distal or total gastrectomy was selected depending on the tumor location. Routine D2 lymph-node dissections were performed according to the Japanese gastric cancer treatment guidelines (fourth edition) (JGCA 2017). Different reconstruction methods, including Billroth I gastroduodenostomy, Billroth II gastrojejunostomy, and Roux-en-Y esophagojejunostomy, were selected based on the extent of gastrectomy. Routine peritoneal cavity washing with at least 1 L of normal saline was performed in both groups after radical surgery. Resected specimens were evaluated by two experienced pathologists to confirm the exact pathological staging. Lymph-node ratio (LNR) was defined as the ratio of the number of metastatic lymph nodes to the number of lymph nodes in the resected specimen (Lorenzon et al. 2014).

II. HIPEC

For the patients allocated to the HIPEC group, two inflow catheters were inserted into the upper abdomen, and two outflow catheters were inserted into the pelvic cavity. The HIPEC treatment was conducted twice within 72 h after gastrectomy. Generally, the first HIPEC treatment was performed within 24 h after surgery followed by the second HIPEC at an interval of 24–48 h. Approximately 3 L of heated normal saline containing cisplatin (40 mg/m^2^) was infused into the peritoneal cavity at a rate of 500 ml/min and was circulated for 60 min using a custom-developed high-precision body cavity hyperthermic perfusion treatment system (BR-TRG-II, Bright Medical Technology Co., Ltd., Guangzhou, China). The temperature of the perfusate was maintained at 43 ± 0.3℃ during the process of intraperitoneal chemotherapy. The perfusate was drained out after the completion of HIPEC.

III. Postoperative systemic chemotherapy

Postoperative systemic chemotherapy based on the SOX regimen (6–8 cycles of S-1 combined with oxaliplatin) was administered to patients in both groups 4–6 weeks after radical surgery. Oxaliplatin (130 mg/m^2^) was administered intravenously on Day 1, and S-1 (80, 100, and 120 mg/day for body surface area below 1.25 m^2^, between 1.25 and 1.5 m^2^ and above 1.5 m^2^, respectively) was administered orally twice a day for 2 consecutive weeks, followed by a 1-week rest.

### Evaluation and follow-up

The postoperative complications were confirmed by the investigators according to the Clavien‒Dindo grading (Dindo et al. 2004), and chemotherapy-related adverse events were evaluated according to the common terminology criteria for adverse events (CTCAE 2010). The OS time was calculated from the date of initial diagnosis to the time of death or the date of the last follow-up. The DFS time was defined as the time from surgery to tumor recurrence.

Follow-up of the entire study population was mainly conducted by telephone and outpatient review. During follow-up, patients underwent physical examination, computerized tomography scans, or serum tumor marker evaluations (including CEA, CA125, CA199, CA242, CA724, AFP, etc.) every 3 months for the first 2 years, and then every 6 months for 3–5 years. The values of these tumor markers were compared with the previous data to detect any potential recurrence or metastasis in advance. The last follow-up was performed in June 2022.

### Sample size

According to some previous studies (Bang et al. 2012; Kang et al. 2021), the 3-year overall survival (OS) rate of AGC patients was 74.2%-83% (mean, 78%). After D2 radical resection with HIPEC and systemic chemotherapy, the 3-year OS rate of AGC patients is estimated to be 86%. Assuming a two-sided α of 0.05 and 90% statistical power, with an estimated dropout rate of 15%, the required sample size was estimated to be 130 patients.

### Statistical analysis

All data were systematically collected to establish a comprehensive database. The data were analyzed by SPSS software for Windows, version 26.0 (SPSS Inc., Chicago, IL, USA). The Chi-square test was used to compare the differences in age, sex, pathologic stage, differentiation degree, tumor size, tumor location, and occurrence rate of metastases. The survival curves were calculated and compared by the Kaplan‒Meier method and the log-rank test. A *P* value < 0.05 was considered statistically significant. Patients without complete data were not included in the final analysis.

## Results

### Patient characteristics

A total of 143 patients were assessed for eligibility, and 134 patients were included and randomly assigned to the HIPEC group and non-HIPEC group at Zhejiang Cancer Hospital from January 2017 to January 2021.

For all patients enrolled, including 104 males and 30 females with a median age of 61 years (22–75 years), there were 118 patients with poorly differentiated adenocarcinoma and 16 patients with moderately differentiated adenocarcinoma. For the entire cohort, the average number of lymph nodes harvested was 33.9 (range 15–76), and the mean LNR was 0.26, while those in the HIPEC group and non-HIPEC group were 36.5 (range 20–76) and 0.25, and 31.3 (range 15–69) and 0.28, respectively. There was no significant difference in the LNR between the two groups (*P = *0.349). According to the postoperative pathologic staging, 13 (9.7%) patients were stage II, while the other 121 (90.3%) patients were stage III. There was no statistically significant difference in sex, age, pathologic stage, or histologic type of tumor between the two groups (Table 1).

**Table 1:** Clinical data of the HIPEC group and the non-HIPEC group

	HIPEC group (*n = *67)	Non-HIPEC group (*n = *67)	*P* value
Age (years)			0.728
≤ 60	31 (46.3%)	29 (43.3%)	
> 60	36 (53.7%)	38 (56.7%)	
Gender			0.407
Male	50 (74.6%)	54 (80.6%)	
Female	17 (25.4%)	13 (19.4%)	
Pathologic T stage			0.770
T3	7 (10.4%)	6 (9.0%)	
T4a	48 (71.6%)	57 (85.1%)	
T4b	12 (17.9%)	4 (6.0%)	
Pathologic N stage			0.529
N0	9 (13.4%)	4 (6.0%)	
N1	9 (13.4%)	11 (16.4%)	
N2	18 (26.9%)	19 (28.4%)	
N3	31 (46.3%)	33 (49.3%)	
Differentiation degree			0.110
Poorly	62 (92.5%)	56 (83.6%)	
Moderately	5 (7.5%)	11 (16.4%)	
Nerve infiltration			1.000
No	19 (28.4%)	19 (28.4%)	
Yes	48 (71.6%)	48 (71.6%)	
Vascular tumor embolus			0.264
No	24 (35.8%)	18 (26.9%)	
Yes	43 (64.2%)	49 (73.1%)	
Tumor size (cm)			0.226
≤ 5	29 (43.3%)	36 (53.7%)	
> 5	38 (56.7%)	31 (46.3%)	
Tumor location			0.511
Cardia	3 (4.5%)	6 (9.0%)	
Body	17 (25.4%)	14 (20.9%)	
Antrum	39 (58.2%)	35 (52.2%)	
Total stomach	8 (11.9%)	12 (17.9%)	
Operation ways			0.662
Open	55 (82.1%)	53 (79.1%)	
laparoscopic	12 (17.9%)	14 (20.9%)	
Surgical procedures			0.481
Distal gastrectomy	42 (62.7%)	38 (56.7%)	
Total gastrectomy	25 (37.3%)	29 (43.3%)	

### Treatment results

For both groups, open and laparoscopic D2 radical resections were performed on 108 and 26 patients, respectively. All enrolled patients achieved R0 resection. Multivisceral resection due to tumor invasion was performed in 4 patients, including 3 patients in the HIPEC group (one patient each had splenectomy, pancreatectomy, and splenectomy combined with left lobe partial hepatectomy) and in 1 patient in the non-HIPEC group (diaphragmatic muscle resection). No operation-related mortality or intraoperative morbidity occurred in either group.

However, postoperative complications regarded as Clavien‒Dindo grade II or above were observed in 11 patients, including 6 patients in the HIPEC group and 5 patients in the non-HIPEC group, without a significant difference. The most common complication was pneumonia (5 cases), followed by intestinal obstruction (2 cases), anastomotic leakage (2 cases), intraperitoneal abscess (1 case) and abdominal hemorrhage (1 case). Resurgery was required in the patient with abdominal hemorrhage for debridement and hemostasis. The other patients suffering from postoperative complications were treated with conservative strategies, and the conditions were well controlled.

In the HIPEC group, 42 (62.7%) patients completed two HIPEC treatments as planned, and the other 25 (37.3%) patients received only one treatment. In both groups, postoperative chemotherapy with the SOX regimen was performed on all patients. A total of 44.8% of patients in the HIPEC group completed at least 6 cycles of postoperative chemotherapy with an average of 4.5 cycles (range 2–8 cycles), while 46.3% of patients in the non-HIPEC group completed at least 6 cycles of postoperative chemotherapy with an average of 4.5 cycles (range 2–8 cycles).

### Treatment toxicity

No adverse events concerning HIPEC treatment were observed in the HIPEC group. Grade 3 or 4 adverse events concerning postoperative chemotherapy were found in 19 patients, including 11 (16.4%) patients in the HIPEC group and 8 (11.9%) patients in the non-HIPEC group, and there was no significant difference between the two groups. Among the whole study cohort, leucopenia/neutropenia (7 patients, 5.2%) and thrombocytopenia (6 patients, 4.5%) were the most common hematological toxic effects, while elevated serum AST levels (11 patients, 8.2%) were the most common nonhematological toxic effect (Table 2).

**Table 2:** Grade 3 or 4 toxic effects in the HIPEC group and the non-HPIEC group

Toxic effects	HIPEC group (*n = *67)	Non-HIPEC group (*n = *67)
Hematological		
Leucopenia/neutropenia	4 (6.0%)	3 (4.5%)
Thrombocytopenia	2 (3.0%)	4 (6.0%)
Non-hematological		
Transaminase elevation	7 (10.4%)	4 (6.0%)

### Survival and recurrence

The median follow-up was 44.0 months (3–65 months). The mean survival time (MST) of the 134 patients was 51.4 months (95% confidence interval [CI] 48.0–55.0 months).

**Fig. 2:**
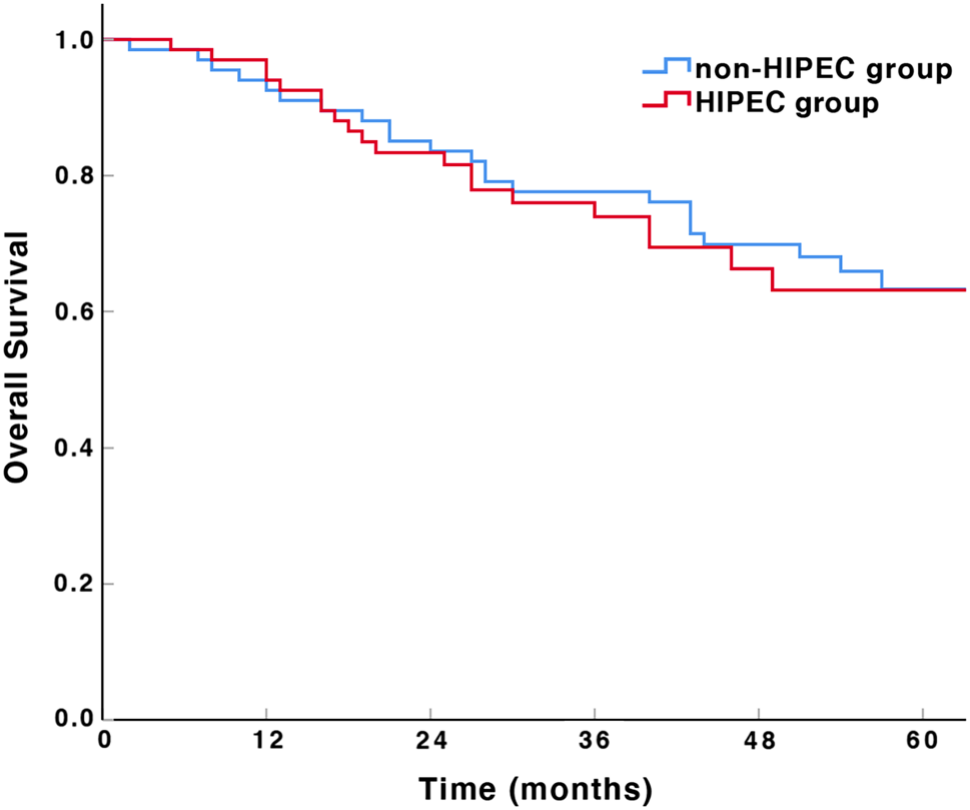
Overall survival of patients with advanced gastric cancer according to different treatments

**Fig. 3:**
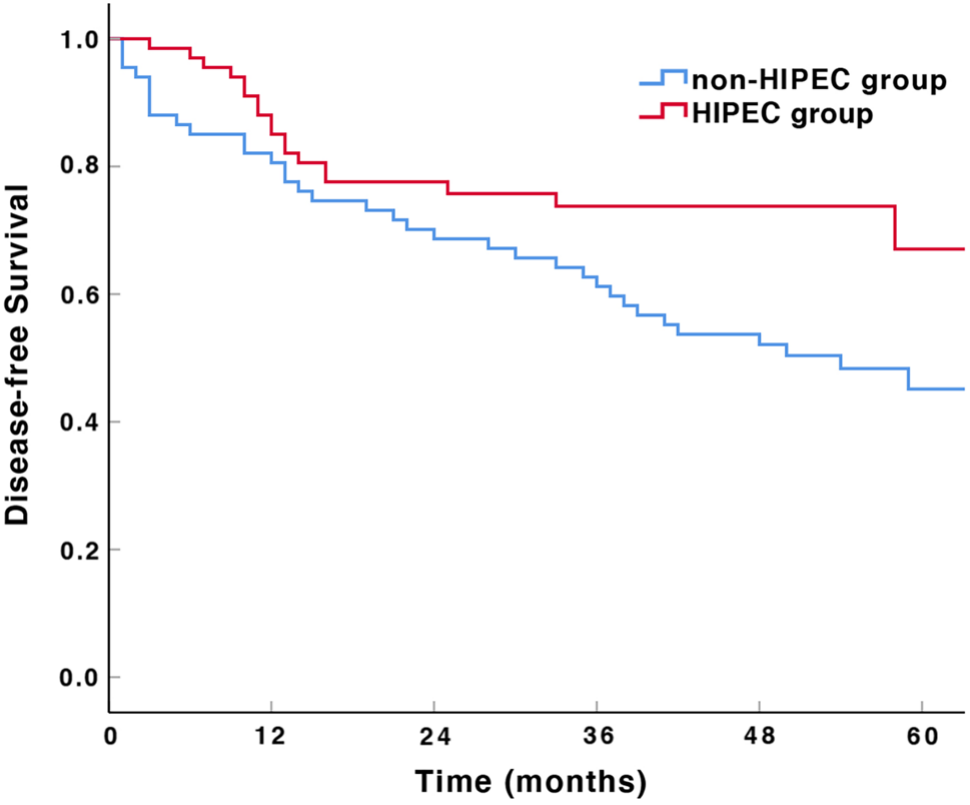
Disease-free survival of patients with advanced gastric cancer according to different treatments

The estimated 3-year OS rates were 73.9% and 77.6% for the HIPEC group and non-HIPEC group, respectively, and the difference was not statistically significant (*P = *0.737, Fig. 2). The 3-year DFS was 73.8% in the HIPEC group and 61.2% in the non-HIPEC group, and the difference was statistically significant (*P = *0.031, Fig. 3).

A total of 53 (39.6%) patients developed distant metastases during the follow-up, including 18 (26.9%) patients in the HIPEC group and 35 (52.2%) patients in the non-HIPEC group, with a statistically significant difference between the two groups (*P = *0.003). Peritoneal metastasis was found in 20.9% (14/67) of patients in the HIPEC group and 40.3% (27/67) of patients in the non-HIPEC group, with a significant difference (*P = *0.015). The other sites of metastases were the liver (1.5% vs. 4.5%), distant lymph node (1.5% vs. 1.5%), lung (1.5% vs. 3.0%), brain (0% vs. 3.0%). and bone (1.5% vs. 0%), and the differences were not statistically significant (Table 3).

**Table 3:** Sites of metastases in the HIPEC group and the non-HPIEC group

	HIPEC group (*n = *67)	Non-HIPEC group (*n = *67)	Total (*n = *134)
Overall	18 (26.9%)	35 (52.2%)	53 (39.6%)
Peritoneum	14 (20.9%)	27 (40.3%)	41 (30.6%)
Liver	1 (1.5%)	3 (4.5%)	4 (3.0%)
Distant lymph node	1 (1.5%)	1 (1.5%)	2 (1.5%)
Lung	1 (1.5%)	2 (3.0%)	3 (2.2%)
Brain	0 (0%)	2 (3.0%)	2 (1.5%)
Bone	1 (1.5%)	0 (0%)	1 (0.7%)

## Discussion

In recent decades, significant improvement has been achieved in the treatment of GC, and the combination of surgery and postoperative chemotherapy is the standard strategy for locally AGC (Bang et al. 2012; Lee et al. 2012; Park et al. 2021). However, the high incidence of PM and the limited effect of systemic chemotherapy are the main reasons for treatment failures. There is a lack of effective management strategies to prevent PM in patients with locally AGC (Dahdaleh and Turaga 2018; Wang et al. 2019; Cortés-Guiral et al. 2021).

Previous studies indicated that HIPEC has an advantage in anti-tumor effects by directly increasing the exposure of free cancer cells to chemotherapy perfusate and enhancing cytotoxicity with the thermo-thermal effect (Verwaal et al. 2008; Desiderio et al. 2017; van Driel et al. 2018). The combination of HIPEC and systemic chemotherapy could effectively improve the prognosis of gastric cancer patients with limited peritoneal metastasis (Yarema et al. 2014; Bonnot et al. 2019; Yu et al. 2020). However, the effect of prophylactic HIPEC in patients with locally AGC remains controversial.

The results of a randomized trial that enrolled 113 GC patients with cT4N0-3M0 indicated that postoperative prophylactic HIPEC plus intravenous chemotherapy could dramatically reduce the possibility of peritoneal recurrence (18.2% vs. 37.9%, *P = *0.020) and improve the DFS and OS rates when the patients with this therapy were compared with patients who did not receive HIPEC treatment (Xie et al. 2020). Our study showed that the 3-year DFS rate of the HIPEC group was better than that of the non-HIPEC group (73.8% vs. 61.2%, *P = *0.031); however, a significant difference in the 3-year OS rate was not observed between the two groups (73.9% vs. 77.6%, *P = *0.737). The results from another meta-analysis, including 13 studies from 1988 to 2021, showed that there was no significant difference in survival rates between the HIPEC group and the control group at the 1-, 2- and 3-year follow-ups, while a statistically significant overall survival effect was found at the 5-year follow-up (Deng et al. 2022). Thus, whether prophylactic HIPEC could effectively improve the long-term survival of locally AGC patients still needs further study. The ongoing PREVENT trial (NCT04447352) and GASTRICHIP trial (NCT01882933) were launched to evaluate the efficacy and safety of HIPEC treatment in locally advanced gastric cancer, with the primary endpoint of OS/DFS/progression-free survival. The results of these studies will partly facilitate the revolution of the combination regimens for HIPEC treatment, which is worth waiting for.

Tumor recurrence and metastasis are common in patients with AGC after radical surgery and the proportion of distant metastasis varies across different studies. Although the proportion of PM is lower in the ACTS-GC (Mitsuru Sasako et al. 2011) and CLASSIC (Bang et al. 2012) studies than in our study, the peritoneum still remains the main site of metastasis. Therefore, an effective treatment strategy for PM is urgently needed to be established. As a regional treatment strategy, HIPEC could effectively eliminate micrometastases and free cancer cells in the abdominal cavity through its thermo-thermal effect. The results from Beeharry et al. showed that the combination of surgery and HIPEC could significantly reduce the peritoneal recurrence rate when compared to surgery alone (23% vs. 3%, *P < *0.05) (Beeharry et al. 2019). In the present study, the occurrence rate of PM in the HIPEC group was significantly lower than that in the non-HIPEC group (20.9% vs. 40.3%, *P = *0.015). Thus, HIPEC was effective in preventing peritoneal metastasis in AGC patients after radical gastrectomy.

However, we found that metastases to other sites, such as liver metastasis and distant lymph-node metastasis, were similar between the two groups, suggesting a limited role of HIPEC in preventing distant metastases other than peritoneal metastases. Therefore, new management strategies should be explored to prevent distant metastasis and improve the prognosis of GC patients. In recent years, chemoimmunotherapy has been widely used in the treatment of patients with locally advanced gastric cancer or metastatic gastric cancer, showing a priority in clinical benefit in preoperative and postoperative settings. (Janjigian et al. 2021; Hasegawa et al. 2022; Kang et al. 2022; Tang et al. 2022). Furthermore, with the rapid development of biomarkers such as Her-2 and CLDN18.2, targeted therapy may play an increasingly important role in managing GC patients (Joshi and Badgwell 2021). However, the clinical benefits of these emerging strategies in preventing distant metastasis need further evaluation in larger sample sizes.

Some studies have reported that HIPEC may increase side effects, such as anastomotic leakage, bowel obstruction, and abdominal sepsis (Verwaal et al. 2008; Mehta et al. 2016). However, Zhang et al. indicated that obvious complications concerning HIPEC were not observed (Zhang et al. 2022). In the present study, there were no serious HIPEC-related adverse events, and the postoperative complications in the two groups were similar. Therefore, it seems that the combination of HIPEC and systemic chemotherapy is a safe and feasible strategy for locally AGC patients who have a high risk of PM.

Although a strict selection criterion was conducted in this prospective randomized study, there are also several limitations. First, this trial was performed in a single center and with a relatively small sample size, which hindered a further comprehensive subgroup analysis. In addition, due to the relatively short duration of follow-up, a significant difference in overall survival was hard to observe. Despite these limitations, the results of this study might be enlightening for future exploration of postoperative adjuvant treatment for locally AGC.

In conclusion, this randomized trial demonstrated that for locally AGC patients with a risk of PM, the adoption of HIPEC combined with systemic chemotherapy could effectively improve the DFS rate and reduce the occurrence of PM without causing serious side effects. However, prospective randomized clinical studies with a large sample size are warranted to validate the results of this study.

## Data Availability

The datasets generated during and/or analyzed during the current study are available from the corresponding author on reasonable request.
